# Whole-genome sequencing guides the successful treatment of extensively drug-resistant TB

**DOI:** 10.5588/ijtldopen.26.0169

**Published:** 2026-09-15

**Authors:** B. Makamure, J. Phelan, T. Bandason, M. Chipinduro, J. Mutsvangwa, J.Z. Metcalfe, F.T. Fernandez, J. Manasa

**Affiliations:** 1 Biomedical Research and Training Institute (BRTI), Harare, Zimbabwe;; 2 London School of Hygiene and Tropical Medicine, London, UK;; 3 Midlands State University, Gweru, Zimbabwe;; 4 University of California, San Francisco;; 5 Bulawayo City Council, Bulawayo, Zimbabwe;; 6 Faculty of Medicine, Laboratory Diagnostic and Investigative Sciences (Medical Microbiology Unit), University of Zimbabwe, Harare, Zimbabwe.

**Keywords:** tuberculosis, Zimbabwe, drug-resistant TB, XDR-TB, TB treatment

Dear Editor,

In 2024, Zimbabwe recorded about 27,000 new TB cases.^1^ Extensively drug-resistant TB (XDR-TB), defined as resistance to rifampicin (R) and isoniazid (H) plus resistance to any fluoroquinolone and either bedaquiline (BDQ) or linezolid (LZD), poses a significant global health challenge.^2^ In 2025, 4.4% of drug-resistant isolates in a study cohort were XDR-TB.^3^ Traditional phenotypic drug-susceptibility testing (pDST) methods often fail to detect the full spectrum of resistance, leading to ineffective regimens and prolonged transmission. Whole-genome sequencing (WGS) provides comprehensive genomic data, identifying resistance mutations, clarifying mechanisms,^4^ and informing personalised treatment strategies, that improve outcomes and strengthen TB control.^5^ Despite its potential, WGS use in Zimbabwe remains limited, due to cost, infrastructure, and technical capacity gaps. We present a Patient scenario where WGS-guided therapy successfully managed a complex case of XDR-TB after multiple treatment failures.

A 29-year-old man, HIV-negative, single, unemployed, and with unknown travel history, was failed by several pre-XDR-TB treatments, and had discordant pDST results (including BDQ and second-line injectables), with limited capacity to assess resistance to other drugs in Zimbabwe. Timeline of baseline diagnostic, pDST results, treatment regimens, monitoring tests, and clinical outcomes are shown in Figure. In April 2018, due to a diagnostic oversight, the patient initially received drug-susceptible TB treatment, and this was corrected in August 2018 to a rifampicin-resistant TB regimen. An administrative error of moxifloxacin (MFX) in January and February 2019 and a stockout of LZD complicated therapy, while discordant BDQ pDST results (2021 vs. 2022) led to BDQ use. Treatment failure resulted from limited tolerance and adherence difficulties. Sputum microscopy and TB cultures were positive in 2019 and chest X-ray revealed new infiltrates (Supplementary Data Figure S1). A new treatment, including intravenous meropenem/clavulanate (MPM-CLAV), was given in 2019–2021 in a hospital facility. MPM-CLAV was stopped after 8 weeks as the patient refused to continue in hospital. Amikacin (AM) was stopped in May 2020. There was initial clinical improvement (5 kg weight increase), but smear and culture positivity recurred at the end of 2020 through 2022, with worsening adherence during COVID-19. In September 2022, the patient committed to directly observed treatment, but outcomes were unsuccessful clinically, radiologically, and microbiologically.

**Figure.**
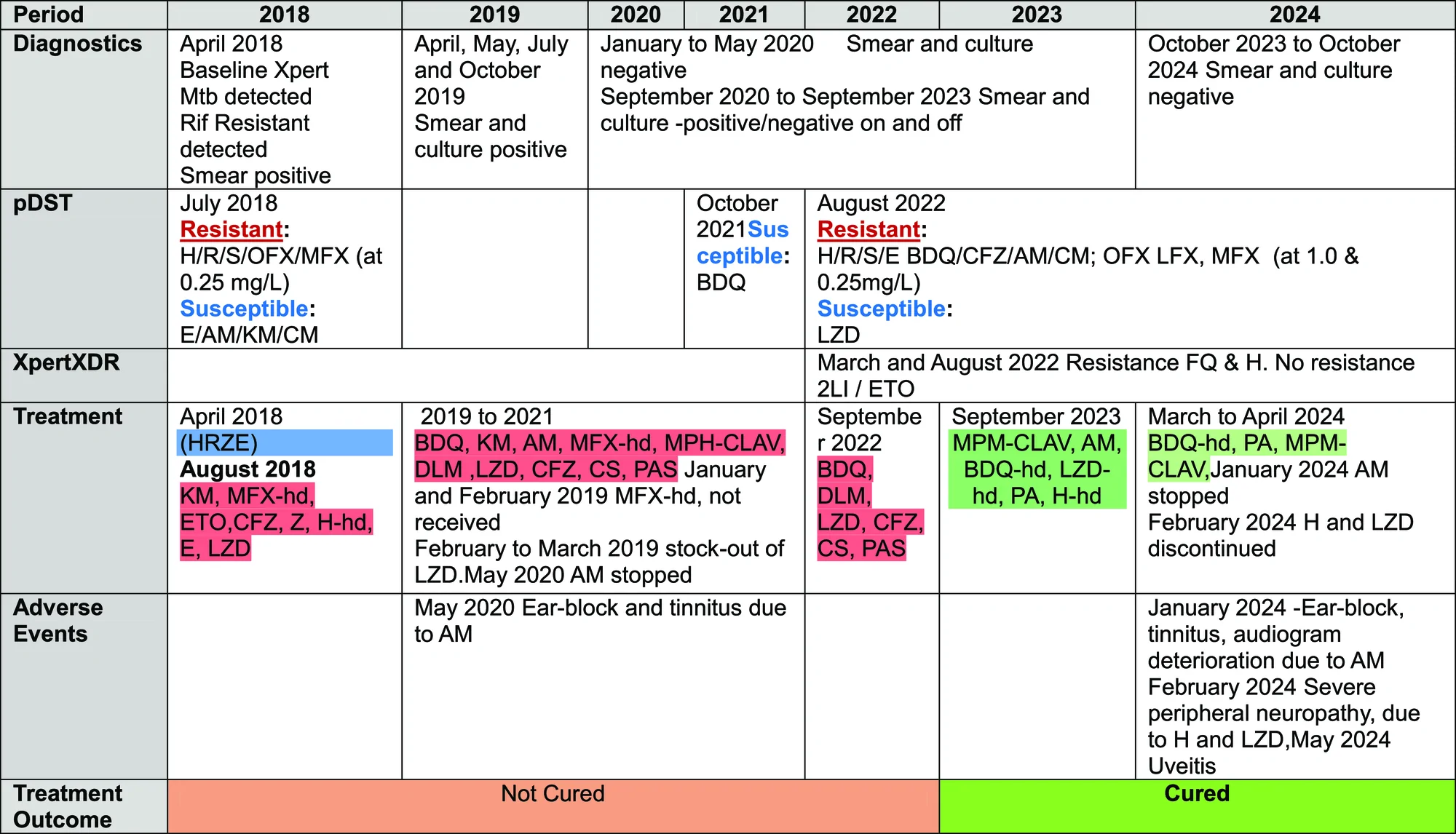
Timeline of diagnostic tests, treatment regimens, and clinical outcomes for a patient with extensively drug-resistant TB in Zimbabwe, 2018–2024. Coloured bars represent treatment phases: blue (first-line), red (second-line), and green whole-genome sequencing–guided treatment). Key adverse events and final cure status are indicated. AM = amikacin; BDQ = bedaquiline; CFZ = clofazimine; CLAV = clavulanic acid; CM = capreomycin; CS = cycloserine; DLM = delamanid; E = ethambutol; FQ = fluoroquinolones; ETO-ethionamide; H = isoniazid; hd = high dose; KM = kanamycin; LFX = levofloxacin; LZD = linezolid; MFX = moxifloxacin; MPM = meropenem; Mtb = *Mycobacterium tuberculosis*; OFX = ofloxacin; PA = pretomanid; PAS = para-aminosalicylic acid; pDST = phenotypic DST; R = rifampicin; S = streptomycin; Z = pyrazinamide; 2LI = 2nd-line injectables.

DNA extracted from longitudinal *Mycobacterium tuberculosis* (Mtb) cultures isolated at the National Tuberculosis Reference Laboratory (NTBRL) in Zimbabwe (2022–2023) was analysed with WGS. The study was conducted at an ISO 15189–accredited Biomedical Research and Training Institute (BRTI) laboratory in Harare, Zimbabwe. Three bio-banked Mtb culture isolates, from the participant, with samples collected in August 2022 (SPC22-00313), October 2022 (SPC22-00462), and July 2023 (SPC23-00398), were resuscitated and DNA extracted, using the cetyltrimethylammonium-bromide method.^6^ The DNA for the first two isolates was shipped by certified international courier (Dalsey, Hillblom, and Lynn, DHL), to the London School of Hygiene and Tropical Medicine (LSHTM) in January 2023 and the third isolate to Borstel, Germany, in September 2023, and sequenced on Illumina platforms. Ethical statement: the samples were part of a study (unpublished, MRCZ/A/2671) examining WGS-determined drug-resistance in Mtb isolates in Zimbabwe. Bio-banked Mtb isolates from previous studies conducted in Zimbabwe and routine TB diagnosis at the NTBRL were used. Participants consented to sample use in other studies. Approval of using leftover samples at the NTBRL, with stripped patient identifiers, to maintain confidentiality, was granted by the Ministry of Health and Child Care. Approvals to conduct the study were obtained from the BRTI Institutional Review Board (AP155/2020), the Parirenyatwa Joint Research Ethics Committee (JREC/183/2021), and the Medical Research Council of Zimbabwe (MRCZ/A/2662). Sample shipment was approved by MRCZ and the Research Council of Zimbabwe.

The LSHTM WGS results received in June 2023 indicated treatment options including AM/KM/capreomycin, delamanid, cycloserine, and LZD. However, a novel variant (p.Val75Gly) in rplC was detected (Supplementary Data Tables S1 and S2). A modified regimen was started in September 2023, with a porta Cath device inserted for better IV access. Subsequent drug adjustments for adverse events followed in January and February 2024. All TB drugs were discontinued for 9 days due to depression to allow the patient a short rest and to visit relatives at home. Treatment resumed in March 2024. A severe left eye uveitis in May 2024 was resolved with topical antibiotics and corticoids. Electrocardiogram-corrected interval using Fridericia’s formula (ECG-QTcF) remained <450 ms, despite receiving high-dose BDQ. By October 2024, after 12 months of negative smears and cultures, the patient was discharged as ‘cured’.

Baseline TB tests diagnosed pre-XDR-TB, but initial mismanagement delayed effective therapy. Discordant BDQ pDST results (2021 vs. 2022) led to an inappropriate regimen including BDQ, potentially amplifying resistance and causing treatment failure. There was high concordance between genotypic and phenotypic DST. WGS results from the UK (July 2023) and Germany (November 2023) showed almost identical results and provided critical insights, identifying resistance mutations, including low-lying and novel mutations missed by pDST, which confer cross-resistance to other antibiotics.

Specifically, WGS results from an international reference laboratory were essential in guiding treatment decisions by identifying the following resistance mutations: 1) Rifabutin (RBT) – RIF had *rpoB_p.Ser450Leu* mutation conferring resistance to RBT,^7^ confirming RBT ineffectiveness for this patient; 2) Mutation *katG_p.Ser315Thr* raised concerns about the efficacy of high-dose isoniazid^8^; 3) Ethionamide (ETO) resistance was linked to *ethA_p.Ala381Pro* limiting ETO activity. Interestingly, no resistance was detected by an Xpert XDR test in August 2022, suggesting a low-level mutation missed by both Xpert and conventional methods.^9^ This limitation underscores the added value of WGS in complex treatment failure scenarios: 4) Fluoroquinolone resistance mutation *gyrA_p.Asp94Gly* rendered MFX ineffective; 5) Additional drug-resistance mutations impacted the effectiveness of pyrazinamide, ethambutol, and streptomycin; 6) Emergent-resistance, a para-aminosalicylic acid (PAS)-related mutation absent in August 2022, was detected in October 2022 sample; 7) *mmpR5_p.Arg134* mutation confirmed resistance to BDQ and clofazimine; 8) LZD-resistance concern, a novel rplC variant, outside the WHO catalogue,^10^ with unknown significance in LZD resistance, highlights the need for exploratory characterisation. Additionally, genome sequencing of a July 2023 sputum culture identified 18 mutations with unknown effects in resistance-related genes, including PAS and LZD. Despite previous findings, LZD remained effective, illustrating the nuanced role of WGS in detecting mutations and guiding treatment. The patient is the first in Zimbabwe to receive prolonged MPM IV (>12 months).

This report highlights the complexity of drug-resistant TB and the necessity of comprehensive genetic analysis for treatment decisions. It demonstrates how WGS can provide critical insights when conventional diagnostic methods fail to detect resistance. It underscores the evolving nature of resistance mutations, emphasising the importance of continuous monitoring.

In conclusion, patients with extensive TB drug resistance are increasingly common in Zimbabwe, requiring clinicians to be well-prepared to manage them. WGS is indispensable for managing complex XDR-TB cases, particularly when pDST is inconclusive for low-lying and novel mutations. We recommend incorporating WGS into the national TB diagnostic algorithms for monitoring treatment failures, research, and control programmes to improve outcomes and work toward ending TB. We also recommend strengthening laboratory and administrative systems to prevent diagnostic and administrative errors, respectively, and proactive resistance monitoring to guide regimen adjustments. The National TB Programme should also avoid drug stockouts.

## Supplementary Material
