# Supplementary material for: Whole-genome sequencing guides the successful treatment of extensively drug-resistant TB
Source: IJTLD Open. 2026 Sep 15;3(9):598–600. doi: 10.5588/ijtldopen.26.0169 (PMC13577212; doi:10.5588/ijtldopen.26.0169)
Supplement: Supplementary file 1 [file ijtldopen26-0169_supplementarydata1.pdf]

Table S1: Whole genome sequencing resistant results for a sputum sample collected in August & October 2022 and July 2023

**Antibiotic**	**Genotypic drug suscep-tibility**	**Mutations detected TAT of results (August and October 2022 samples) was 5 months and for the July 2023 sample was 3 months**					
		**August 2022 (SPC22_00313)**	**October 2022 (SPC22_00462)**	**July 2023SPC2300398 (INT36)**	**Confidence**	**Mutations with unknown effect in resistance implicated genes**	**Frequency**
Isoniazid (H)	R	katG_p.Ser315Thr	katG_p.Ser315Thr	katG S315T	High	mshA A254G	5.83
Rifampicin (R)	R	rpoB_p.Ser450Leu	rpoB_p.Ser450Leu	rpoB S450L	High	ponA1 P631S	97.03
Ethambutol (E)	R	embB_p.Met306Ile	embB_p.Met306Ile	embB M306I	High	EmbB S174R	5.52
Pyrazinamide (Z)	R	pncA_p.Cys14Arg	pncA_p.Cys14Arg	pncA C14R		Rv3169 A190G<br>Rv3169A194P	26.00<br>27.78
Streptomycin (S)	R	gid_p.Leu79Ser,<br>rrs_n.514A>C	gid_p.Leu79Ser,<br>rrs_n.514A>C	rrs 514A>C	Low	gid L79S	100.00
Levofloxacin (LFX)	R	gyrA_p.Asp94Gly	gyrA_p.Asp94Gly	gyrA D94G	High	eccC5 L1115H	5.29
Moxifloxacin (MXF)	R	gyrA_p.Asp94Gly	gyrA_p.Asp94Gly	gyrA D94G	High	eccC5 L1115H	5.29
Ofloxacin (OFX)	R	gyrA_p.Asp94Gly	gyrA_p.Asp94Gly	gyrA D94G	High	eccC5 L1115H	5.29
Ethionamide (ETO)	R	ethA_p.Ala381Pro	ethA_p.Ala381Pro			mshA A254G<br>ethA A381P	5.83<br>100.00
Bedaquiline (BDQ)<br>Clofazimine (CFZ)	R<br>R	mmpR5_p.Arg134*	mmpR5_p.Arg134*	Rv0678 140_ins_tc<br>Rv0678 R134_	Low<br>High	Rv1979c R409Q	99.77
Ciprofloxacin (CFX)	R	gyrA_p.Asp94Gly	gyrA_p.Asp94Gly		High	-	-

Key: R – resistant, absence of a mutation from the interpretation catalogue of the Research Center Borstel (v20221122). High and Low confidence determination is from the WHO catalogue of mutations. TAT – Turnaround time

Table S2: Whole genome sequencing susceptible results for a sputum sample collected in August & October 2022 and July 2023

Antibiotic	Sequence result	Mutations detected					
		August 2022 (SPC22_00313)	October 2022 (SPC22_00462)	July 2023 SPC23-00398 (INT-36)	Confidence	Mutations with unknown effect in resistance implicated genes	Frequency
Rifabutin (RFB)	-	-	-	-	-	ponA1 P631S	97.03
Compensates for rpoB mutations	-	-	-	-	-	rpoC V483G	99.53
Amikacin (AM)	S	-	-	-	-	-	-
Kanamycin (KM)	S	-	-	-	-	-	-
Capreomycin(CM)	S	-	-	-	-	-	-
Gatifloxacin (GFX)		-	-	-	-	eccC5 L1115H	5.29
Cycloserine (CS)	S	-	-	-		PPE22 V288G<br>3841652T>A<br>3841654T>G<br>3841663C>T	6.23<br>18.74<br>19.10<br>97.52
Prothionamide (PTO)	S	-	-	-	-	mshA A254G<br>ethA A381P	5.83<br>100.00
Para-aminosalicylic_ acid (PAS)		-	thyX_c.-16C>T	-	-	3074495G>A	71.99
Linezolid (LZD)	S	-	-	-		rplC V75G	64.42
Delamanid (DLM)	S	-	-	-	-	-	-
Thioacetazone (T)		-	-	-	-	739262C>T<br>ethA A381P	99.89<br>100.00
Beta-lactams	-	-	-	-	-	ponA1 P631S	97.03
compensates for ThyA deficit	-	-	-	-	-	3067961G>A	28.35

R - resistant, S - susceptible, - absence of a mutation from the interpretation catalogue of the Research Center Borstel (v2022-11-22)

Figure legends

Figure S1: Patient chest X-rays showing clinical evolution from 2018 to 2020

Figure S1

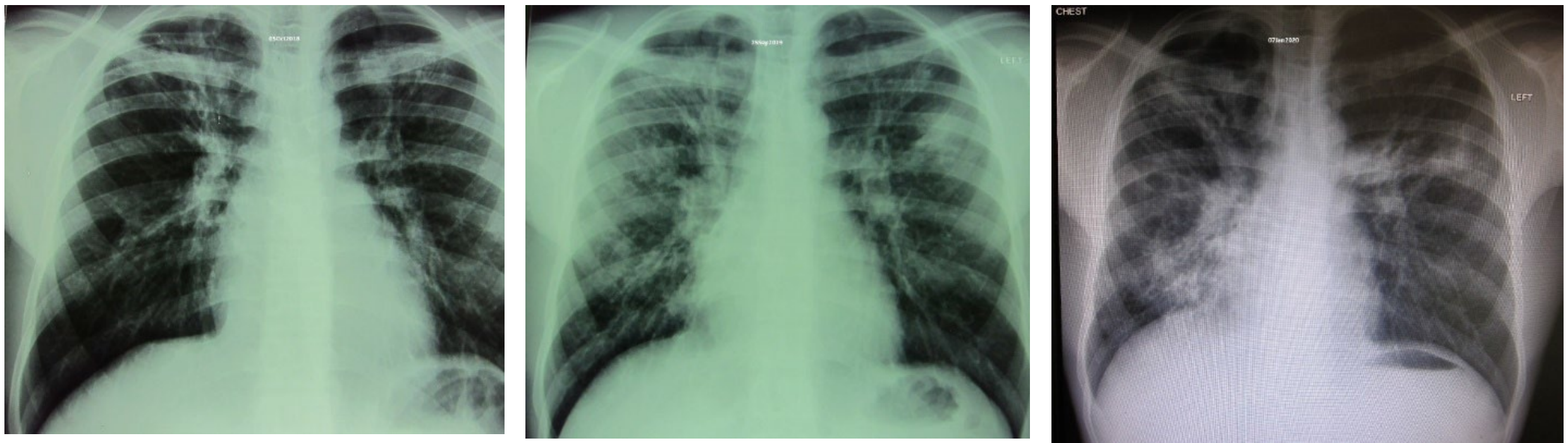

5 October 2018 **25 September 2019** **7 January 2020**
